# Clinical Cases

**Published:** 1883-04

**Authors:** E. W. Berridge

**Affiliations:** London


### CLINICAL CASES.

E. W. Berridge, M. D., London.

(1) *Lachesis in carbuncles.* May 22d, 1876.—About a week ago Mr. S. applied an ointment to a slight eruption on right tibia; erysipelas, with pustules, ensued; then the affected part of the leg became swollen *bluish-red*, and discharged pus from several holes, presenting the appearance of a large carbuncle, with itching. *Lachesis*$^{cm}$ (Fincke) every three hours.

May 23d.—Less inflamed; continue medicine.

May 26th.—Better; less itching, but still blue; continue medicine.

June 1st.—Has steadily improved. For the last few days has taken *Lachesis*$^{mm}$ (Bœricke) every four hours. Carbuncle now healing, less blue, looks red around edges; a core has come out of the holes. Continue medicine.

June 7th.—Much better. The carbuncle has now become an open wound, which is healing. Stop medicine.

June 30th.—Wound healed. Can bear pressure on it.

April, 1877.—No return of symptoms.

(2) *Kali carb.* Jan. 24th, 1876.—Miss B. complained that for thirteen days she has had an enlarged gland in right axilla, with aching, burning pain in it and also shooting to ends of right fingers, to left hypochondrium, to inner edge of left scapula, and to left breast; the enlarged gland itches. Guided by Jahr's German *Repertory* (vol. ii, p. 963), I selected *Kali carb.*, and gave a dose of 4M (Jenichen) three times a day.

Jan. 26th.—Better since yesterday; itching still; the pain lessened on evening of 24th, and is nearly gone to-day. Swelling gone. She took the remaining five or six globules, a dose morning and evening, and in two or three days was quite well and remained so. Probably one dose would have cured the case.

Will any adherent of the pathological school explain the pathology of the above case and point out on pathological principles why *Kali carb.* cured, and also show, if possible, how I could have made a better cure by forsaking the rules of Hahnemann and prescribing according to the more "scientific" (!?) Hughesian method?

(3) *Hydrophobinum.* Miss S. has had yesterday and to-day constant desire to urinate *on seeing running water*, urinating only a little at a time. At 12.40 P. M. I gave her one dose of *Hydrophobinum*$^{cm}$ (Swan). Within an hour she was much better, and soon recovered.

This case, which verifies several similar ones recorded in our literature, proves that the symptoms produced by a crude disease-virus may be incorporated with the provings and derived symptoms of the corresponding dynamized nosodes, as Hering has done in two cases of *Anthracinum*, provided we can ascertain that the symptoms are really due to the virus alone, which is not always an easy task.

(4) *Belladonna in toothache.* Mr. W. for fourteen days has had pain in first left upper molar, *suddenly coming and going*, dragging downward. During the pain he is sleepy and low-spirited. The pain is paroxysmal, lasting about ten minutes, with intervals of about twelve hours on an average. He has now (11 P. M.) just recovered from an attack. I gave him a dose of *Bellad.*$^{dm}$ (Swan's fluxion), and told him to repeat the dose after each attack. During the next day had a slight attack morning, afternoon, and evening; during the next a slight attack morning and afternoon. After this, no return of the pain. Took only two doses. He told me that this cure had impressed him with the power of homœopathic remedies more than anything he had ever before experienced.

(N. B.—By "Swan's fluxion" I mean some very high potencies prepared by using a *continuous* stream of water *from first to last*, and not by potentizing on a fraction of a drop, as described in *The Organon* as his later method.)

(5) *Mercurius in ulcerated throat.* December 15th, 1875.—Miss C. caught cold on evening of 11th. Sore throat commenced on 12th. Has now pricking in right side of throat on swallowing anything; right side of throat feels swollen, hot, and dry; right tonsil inflamed and ulcerated. Pulse, 108, weak and intermittent. *Cold air hurts throat.* Tongue thickly coated, white. *Merc. vivus*$^{10m}$ (Fincke) every two hours.

Dec. 17th.—Very much better. Tongue cleaning at edges; stronger; throat better; swallows better. Continue medicine every three hours.

Dec. 18th.—Throat well; tongue white only in centre. Stop medicine.

Dec. 20th.—Quite well, except that the appetite is not quite so good as usual.

(6) *Mercurius in vaginitis.* Nov. 15th, 1875.—Miss S., æt 40. For seven days burning, throbbing, and itching in vagina and external genitals, especially the latter; worse on left side. For some weeks numbness down left leg. For three months swelling in hypogastrium. Abdomen swollen and feels hard internally. Yellow discharge from vagina. On mucous surface of genitals, reaching as high up as she could see, and on the external parts, especially near urethra, are whitish patches like aphthæ, and when they are removed a red, raw surface is left, uneven to touch, and with little red elevations on it. The throbbing is relieved by lying still. The burning is worse from cold and from washing, especially cold washing. For a week has not been able to go to closet on account of the draught of air increasing the pain, but had to use the chamber utensil instead. The throbbing, burning, and itching are worse if she thinks about them, and thinking about the pains also makes her want to urinate. Menses delayed a week, and previous to their last appearance delayed eleven months. For seven days the contact of the urine has made the parts burn. For four days thrill in urethra when the urine passes.

According to the characteristics given at p. 572 of Guernsey's *Obstetrics*, I selected *Mercurius vivus*, and gave a dose of 10M (Fincke) every three hours.

Nov. 16th.—Better after second dose. Menses appeared *after* the improvement had commenced. Continue medicine.

Nov. 17th.—Still better. *Mercurius vivus* $^{mm}$ (Swan's fluxion) every three hours—the first dose at 5 P. M. I gave a high potency because the 10M, though it relieved, did not act with that rapidity which I had almost always found high potencies act on this patient. All the evening after this first dose of the higher potency was quite free from pain, and had only the itching; she did not feel such marked relief from 10M. Took another dose at 11 P. M. and felt pretty well all night.

Nov. 18th.—Took another dose at 10 A. M. To-day, costive. At 1 P. M. no throbbing; slight return of burning; less itching; scalding of urine decidedly relieved; swelling of abdomen continues. Menses continue. Less aggravation from cold air when at stool since the higher potency; thrill in urethra gone; numbness in left leg still; says the last potency has done her more good than the former. As she understood Hahnemannian Homœopathy, far better, indeed, than most "physicians practicing Homœopathy," I gave her some more globules of the higher potency to take if needed.

Feb. 1st, 1876.—Sent me the following report: The acute symptoms all ceased, returning in two days, though less severely, ceasing again without repetition of the dose. On Nov. 25th, they returned and did not decrease again as before, so she took another dose of the higher potency, which removed them completely After the last dose the swelling in hypogastrium and the swelling and hard feeling in abdomen gradually ceased, disappearing later than the other symptoms; the numbness of left leg ceased after the higher potency, but before the last dose was taken; the discharge was also relieved. Up to to-day there has been no return of any of the symptoms except that a slight discharge continues. As she subsequently had to take other medicines the effect of *Mercurius* on the discharge was complicated, but from the relief it gave I believe it would have cured it.

*Comments:* (1) Hahnemann recites (*Organon*, 287, note) that the higher the potency, the more rapid and penetrating is the action. This case verifies his statement; the MM potency did far more than the 10M. I have seen *Phosphorus*$^{mm}$ (Fincke) rapidly relieve a cough after CM (Fincke), given for two days, had done nothing. In a chronic case, where *Sulphur*$^{2m}$ (Jenichen) and CM (Fincke) had done nothing, successive doses of MM (Bœricke) and 10MM and 20MM (Swan's fluxions) greatly relieved, the completeness and duration of the relief being in proportion to the height of the potency. These are *facts*, which the sneers of those who fail because they do not know how to select their remedies can never overthrow.

(2) This case also verifies Hahnemann's teaching—that in a truly homœopathic cure symptoms disappear in the inverse order of their appearance.

(3) It confirms, also, some of Guernsey's key-notes, and adds some important clinical symptoms for future verification, especially with regard to the mental conditions.

(4) The aggravation of the pains from cold air is a new symptom, which I cannot find either in the provings or recorded clinical experience; but it is analogous to the similar aggravations of throat symptoms, also not in the provings, but given by Bœnninghausen, and verified by myself in the preceding case. This shows that a perfect Repertory or Materia Medica must contain *collectives* of conditions, etc., that we may select our remedies by analogy when necessary.

(5) When the symptoms, having ceased, returned with less severity, the patient did right in not repeating the dose. Had she done so, an aggravation might have occurred. When, however, they afterward returned in the same form, *and persisted*, the dose was repeated with benefit.

(6) The relief of the acute symptoms *preceded* the re-establishment of the menses, though they had existed exactly the same time. This shows that it was a homœopathic cure. Had they disappeared in the reverse order, it would have shown that it was merely a natural recovery.

